# Ultrasound guided fine needle aspiration biopsy of parathyroid gland and lesions

**DOI:** 10.1186/1742-6413-3-6

**Published:** 2006-03-28

**Authors:** Haytham Dimashkieh, Savitri Krishnamurthy

**Affiliations:** 1 Departments of Pathology, The University of Texas M. D. Anderson Cancer Center, Houston Texas, USA

## Abstract

**Background:**

Parathyroid gland and their tumors comprise a small proportion of non-palpable neck masses that are investigated by ultrasound (US) guided fine needle aspiration biopsy. We reviewed our institution's cases of US guided FNAB of parathyroid gland and their lesions to determine the role of cytology for the preoperative diagnosis of parathyroid gland and their lesions.

**Method:**

All cases of FNAB of parathyroid gland and lesions in the last 10 years were reviewed in detail with respect to clinical history and correlated with the histopathologic findings in available cases. The cytologic parameters that were evaluated included cellularity assessed semiquantitatively as scant, intermediate or abundant (<50, 51–500 or >500 cells), cellular distribution (loose clusters, single cells/naked nuclei, rounded clusters, two- and three-dimensional clusters, and presence of prominent vascular proliferation), cellular characteristics (cell size, nuclear shape, presence/absence of a nucleolus, degree of mitosis, amount of cytoplasm, and appearance of nuclear chromatin), and background (colloid-like material and macrophages). Immunostaining for parathyroid hormone (PTH) was performed on selected cases using either destained Pap smears or cell block sections.

**Results:**

Twenty cases of US-guided FNAB of parathyroid glands and their lesions including 13 in the expected locations in the neck, 3 in intrathyroid region, 3 in thyroid bed, and 1 metastatic to liver were studied. Majority of the cases showed intermediate cellularity (51–500 cells) with round to oval cells that exhibited a stippled nuclear chromatin, without significant pleomorphism or mitotic activity. The cells were arranged in loose two dimensional groups with many single cells/naked nuclei around the groups. Occasionally macrophages and colloid like material was also encountered. There was no significant difference in the cytomorphologic features between normal gland, hyperplasia adenoma, or carcinoma. Immunocytochemical analysis for PHT was performed for 14 cases (6 destained smears and 8 cell blocks) which showed distinct cytoplasmic positivity.

**Conclusion:**

US-guided FNAB is a useful test for confirming the diagnosis of not only clinically suspected parathyroid gland and lesions but also for detecting parathyroid glands in unexpected locations such as in thyroid bed or within the thyroid gland. Although there is significant overlap in the cytomorphologic features of cells derived from parathyroid and thyroid gland, the presence of stippled nuclear chromatin, prominent vascular proliferation with attached epithelial cells, and frequent occurrence of single cells/naked nuclei are useful clues that favor parathyroid origin. Distinction of the different parathyroid lesions including hyperplasia, adenoma, and carcinoma cannot be made solely on cytology. Immunostaining for PTH can be performed on destained Pap smears and cell block sections which can be valuable for confirming parathyroid origin of the cells.

## 

Parathyroid glands and their tumors comprise a small proportion of non-palpable neck masses that are investigated by ultrasound (US)-guided fine-needle aspiration biopsy (FNAB). Patients with signs and symptoms of hyperparathyroidism are not usually evaluated preoperatively using US-guided FNAB if enlargement of the gland is confirmed sonographically. However, when the parathyroid gland and parathyroid lesions are located in unexpected sites, they are typically investigated using US-guided FNAB. Awareness of the atypical locations of parathyroid glands and recognition of cytomorphologic features is important to avoid misdiagnosis of such cases. Very few studies have reported the utility of US-guided FNAB in diagnosing these lesions preoperatively [1-4]. In addition, conflicting reports have appeared regarding the cytomorphologic features of parathyroid lesions [3,5-10]. We reviewed our institution's cases of US-guided FNAB of parathyroid gland and their lesions to determine the role of cytology for the preoperative diagnosis of parathyroid glands and their lesions.

## Materials and methods

We reviewed cytology reports filed at The University of Texas M. D. Anderson Cancer Center between January 1995 and January 2005 to identify cases for which parathyroid origin of the specimen was indicated in the cytology report. The patients' demographic data and clinical histories were recorded in detail. The available histopathology slides of cases in patients who subsequently underwent surgery were reviewed and their features were correlated with the cytology findings. The cytology smears were examined in detail and several cytologic parameters were studied. These parameters included cellularity assessed semiquantitatively as scant, intermediate, or abundant if there were <50, 51–500 or >500 cells in the smear, cellular distribution (loose clusters, single cells/naked nuclei, rounded clusters, two- and three-dimensional clusters, and presence of prominent vascular proliferation in the clusters), cellular characteristics (cell size, nuclear shape, presence/absence of a nucleolus, degree of mitosis, amount of cytoplasm, and appearance of nuclear chromatin), and background e.g., presence of colloid-like material, macrophages.

US-guided FNA was performed by the radiologist with a 7.5 to 10-MHz transducer, using a 23- or 25-gauge needle attached to a 10-ml syringe. Direct smears were fixed in 95% alcohol for Papanicolaou staining and air dried for Diff-Quik staining. In each case, the syringe was then rinsed in RPMI, and the cells rinsed out of the syringe were used to make either a cytospin slide or a cell block, depending on the size of the cell pellet produced after centrifugation. Cytospin slides were stained with Papanicolaou stain, and cell blocks were fixed in formalin, embedded in paraffin, cut into 5 μm thick sections, and stained with hematoxylin and eosin.

Immunostaining for parathyroid hormone (PTH) was performed on selected cases using either Pap smears or cell block sections. The Pap smears were destained with 1% acid alcohol prior to immunostaining. Immunostaining was performed in a Dako autostainer by the avidin-biotin-peroxidase technique using PTH (DakoCytomation, Carpinteria, CA; 1:50 dilution) as the primary antibody and 3-amino; 9 ethyl-carbazole (AEC) as the chromogen.

## Results

**Figure 1:**
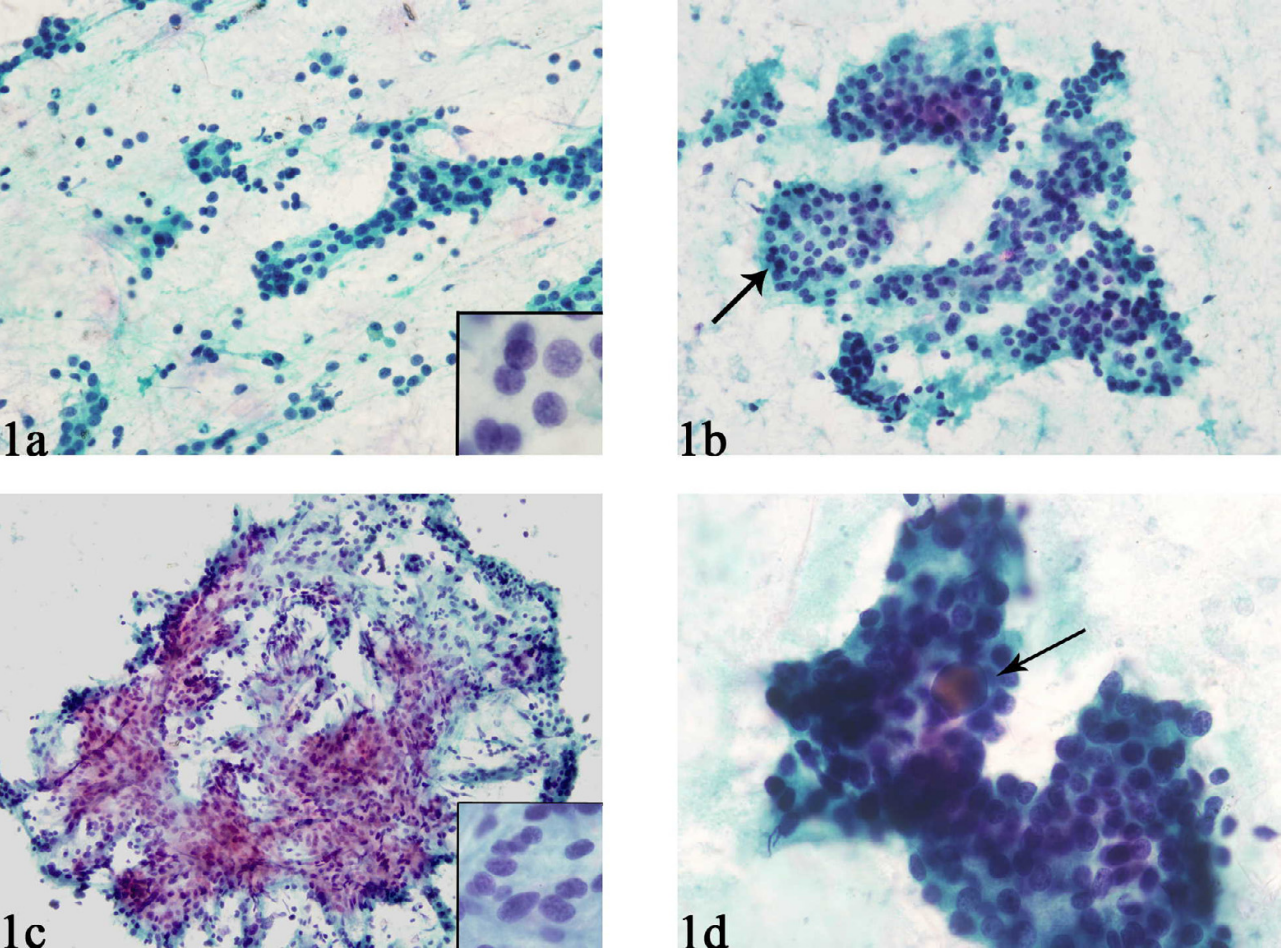
Common and uncommon cytomorphologic features of fine-needle aspiration biopsy (FNAB) smears of parathyroid gland. (A) FNAB smears commonly showed cells arranged as loose clusters with many single cells and naked nuclei in the background. The nuclei are small, round, and have stippled nuclear chromatin (inset). (B) Sometimes the clusters had a rounded configuration, mimicking thyroid follicles (arrows). (C) A prominent vascular network was sometimes apparent. Mild nuclear pleomorphism was sometimes evident (inset). (D) Uncommonly, colloid-like material was present within the cluster of cells (arrow).

Twenty cases for which US-guided FNAB was performed on parathyroid glands and their lesions (13 in the expected locations in the neck, 3 in intrathyroid region, 3 in the thyroid bed, and 1 metastatic to the liver) were identified and studied. In 17 cases smears were available for review, while in 3 cases only cell block material was available; in 12 cases both smears and cell blocks were available. The cytomorphologic findings from the smears are summarized in Table 1. Overall the cellularity ranged from scant (< 50 cells) to abundant (> 500 cells), with the majority of the cases having intermediate numbers (51–500 cells). The most consistent nuclear features of the parathyroid cells were the presence of stippled chromatin and their round to oval shape (Fig. 1A, 1B). Only a few cases had significant pleomorphism (n = 3) (Fig. 1C), 1 case had nucleoli, and no mitoses were present in any case. The parathyroid cells were predominantly arranged in loose 2-dimensional groups, which often was round, mimicking thyroid follicles (Fig. 1B). Sometimes the cluster was small, resembling a microfollicle (n = 6). Three-dimensional groups were also present in 2 cases. A prominent vascular network was evident in 6 cases, with epithelial cells clinging to the vessels (Fig. 1C). Single cells, either with a thin rim of cytoplasm or appearing as naked nuclei, in many cases appeared near larger groups of cells (Fig. 1A). Macrophages were present in the background in 3 cases, and inspissated colloid-like material was present in 2 cases. The colloid-like material was found either embedded within the cells or distributed separately in the vicinity of the cells (Fig. 1D). There was no significant difference between Pap and Diff-Quik stained smears.

**Table 1:** Cytomorphologic Features of US-Guided FNAB of Parathyroid Glands/Lesions.

	**n**	**%**
**Nuclear features**		
Nucleolus	1	6
Mitosis	0	0
Stippled chromatin	17	100
Pleomorphism	3	18
Round to oval shape^a^	16	94
**Cell arrangement**		
Single cells/naked nuclei	9	53
Loose 2-dimensional clusters	16	94
3-dimensional clusters	2	12
Rounded clusters	5	29
Vascular proliferation	6	35
**Background**		
Macrophages	3	18
Colloid-like material	2	12

^a^Nuclear shape was round to oval in all cases, except in the case of metastatic parathyroid carcinoma to liver, where the nuclei were oval and spindle in shape and also showed vascular proliferation.

**Figure 2:**
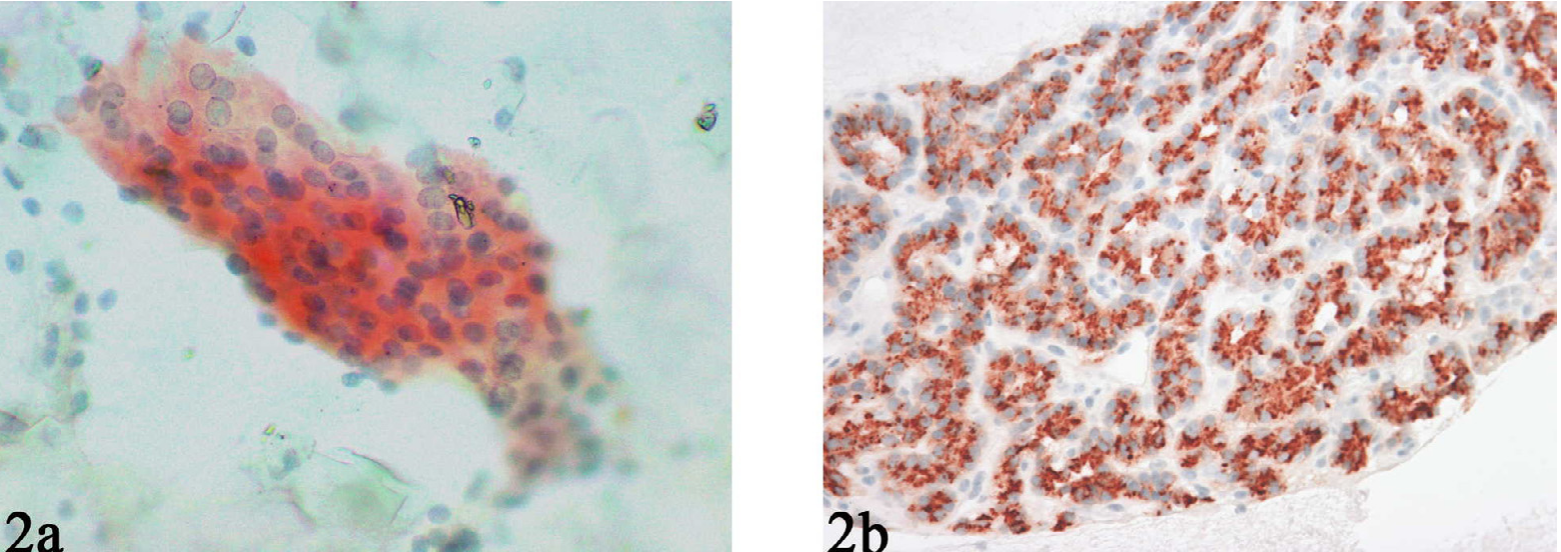
Parathyroid hormone (PTH) immunostaining performed on a destained Pap smear (A) and cell block (B) showing distinct cytoplasmic positivity.

Immunocytochemical analysis for parathyroid hormone (PTH) was performed for 14 cases (6 destained smears and 8 cell blocks) and showed distinct cytoplasmic positivity (Fig. 2).

Cytologic-histologic correlation of the cases that were diagnosed as parathyroid tissue on cytologic analysis were interpreted on surgical excision as hyperplasia in three cases, adenoma in ten cases, and carcinoma in three cases. The smears showed no significant variability in cytomorphologic features amongst these cases. Therefore we did not find any cytomorphologic feature that suggested or represented a particular lesion.

Table 2 summarizes the presence of hypercalcemia/hyperparathyroidism relative to the anatomic location of all the cases except the case of metastatic parathyroid carcinoma to liver. Among the 13 cases with the lesion at the expected location in the neck, 10 patients had hypercalcemia. Five of these patients had a history of hypercalcemia/hyperparathyroidism and FNAB had been performed to rule out recurrent disease; in the other 5 cases, the patients had presented for the first time with hypercalcemia/hyperparathyroidism and FNAB was performed to localize the lesion. In 3 of the 13 cases the patients presented with an incidental neck mass without hypercalcemia, and thus FNAB was performed to determine the nature of the lesion. In the 3 cases of intrathyroidal parathyroid lesions, all the patients presented with hypercalcemia and radiographic diagnosis was of a thyroid mass. Among the 3 thyroid bed parathyroid glands/lesions, 2 had FNAB performed to rule out recurrent disease (1 patient had a history of papillary thyroid carcinoma, and the other a history of medullary thyroid carcinoma); the remaining patient, who presented with hypercalcemia and a left thyroid bed mass, had a history of left hemithyroidectomy.

**Table 2:** Sites of US-Guided FNAB of Parathyroid Glands/Lesions.

	**ANATOMIC LOCATION**		
**Clinical presentation**	**Expected site (Neck)**	**Intra-thyroid**	**Thyroid bed**
^a^Hyperca/HyperPTH +	10	3	1
Hyperca/HyperPTH -	3	0	2
Total	13	3	3

^a^The case of metastatic parathyroid carcinoma to the liver was excluded. Hyperca: hypercalcemia; HyperPTH: hyperparathyroidism.

**Figure 3:**
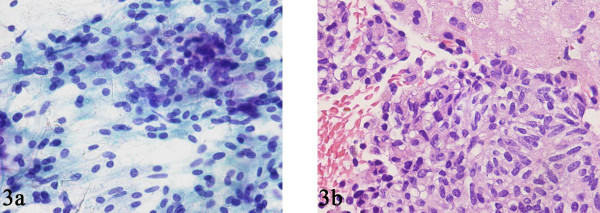
FNAB smear (A) and core biopsy (B) specimens from a case of metastatic parathyroid carcinoma in the liver showing sheets of relatively bland spindle and round cells.

We had only 1 case of metastatic parathyroid carcinoma to the liver in our study. The smears showed spindle and round cells with a relatively bland cytology and minimal atypia that were arranged in 2-dimensional sheets interspersed with a delicate vascular network (Fig. 3). The nuclear chromatin was finely granular, and mitotic activity was insignificant. Single cells/naked nuclei were not frequent. The cell block sections showed the spindle and round cells to have a pale to clear cytoplasm (Fig. 3). Immunohistochemical staining for PTH revealed strong cytoplasmic positivity in the tumor cells, which was instrumental in confirming the diagnosis of metastatic parathyroid carcinoma.

## Discussion

Parathyroid glands can vary in number and location in normal subjects. Most people have 4 glands, which are located posterior to the upper and lower poles of the thyroid gland. However in as many as 25% of normal subjects, more than 4 glands are present [11,12]. The inferior parathyroid glands are much more variable in their distribution than the superior glands [13]. Inferior parathyroid glands may be located in the lower pole of the thyroid or in thymic tongue or they may be juxtathyroidal or mediastinal [14]. Normal parathyroid glands are usually not detectable on any imaging modality because of their small size and a structural pattern that is very similar to that of thyroid parenchyma. However, when there is clinical evidence of hyperparathyroidism, high-frequency sonography is an accurate and non-invasive means of localizing the enlarged parathyroid glands. Preoperative localization of parathyroid tumors is highly recommended since it reduces operative time, especially when the glands are in abnormal locations and reduces the risk of damage to the laryngeal nerve and normal parathyroids [15-17]. Preoperative tumor localization is especially important in patients who have had previous neck surgery which can produce scarring and distortion of the anatomic landmarks and thus complicate subsequent exploration [18,19]. Ectopic parathyroid tumors are difficult to detect sonographically; intrathyroidal glands can mimic thyroid nodules, and undescended glands situated along the course of the common carotid artery or recurrent laryngeal nerve can resemble lymph nodes [20]. The use of preoperative FNAB can increase the accuracy of parathyroid gland localization [1-3,5,18,21,22]. Therefore, preoperative FNAB is very often used for locating suspected parathyroid tissue in patients for whom previous exploratory surgery was unsuccessful but who have remained hypercalcemic,^1 ^to determine ectopic locations of suspected parathyroid lesions, [1,23] particularly when the sonographic findings are not conclusive [22]; and in cases of parathyroid lesions presenting as cystic masses [24].

In our study, the aberrant anatomic localizations of parathyroid glands/lesions included intrathyroid region and thyroid bed. Most lesions, however, occurred in the expected locations in the neck. FNAB, were performed either to confirm the presence and location of parathyroid tissue in cases where clinical and radiographic data suggested hyperparathyroidism or to determine the nature of the neck mass in patients undergoing staging workup for thyroid malignancy.

The cytomorphologic features of parathyroid glands have been described by few investigators who used either touch preparations or FNA of surgical specimens [25,26] and preoperative FNAB materials [1-4]. Our findings with regards to the cytomorphologic features of parathyroid cells are very similar to the studies and case reports reported in the literature [1-4,6-8,25,27-30]. We agree with the majority of the previous reports that distinguishing parathyroid cells from thyroid follicular cells is the most difficult aspect of parathyroid cytology. We found that in comparison to thyroid follicular cells, parathyroid cells are smaller and have less cytoplasm. In addition, the nuclei of the parathyroid cells are usually round to oval with stippled nuclear chromatin (what is commonly referred to as a salt-and-pepper appearance). However, nuclear pleomorphism was encountered occasionally and a few atypical larger nuclei were observed. While nucleoli were usually inconspicuous, they were prominent in rare cases. Mitotic activity was generally insignificant. Parathyroid cells had pale scant cytoplasm; the cytoplasm sometimes contained vacuoles with ill-defined borders. Shidham et al have emphasized the value of Romanowsky staining (Diff-Quik) in highlighting intracytoplasmic fat vacuoles in their study of the utility of intraoperative imprint cytology for the confirmation of various tissues in the parathyroid region [31]. They found round, oval and clear vacuoles with a sharp outline and with a tendency to indent the nucleus more often in Romanowsky stained cells from direct smears of normal Parathyroid glands in comparison to those with hyperplasia and adenoma. In addition they also found granular and lacy background owing to ruptured cytoplasm of the cells and absence of coarse paravacuolar granules to be a useful features for the distinction of cells of Parathyroid origin from those of thyroid origin. Unlike Shidham et al we did not find the cytomorphologic features of parathyroid cells on Diff- Quik smears to be very different from those noted on Pap smears. It is to be noted that while they studied imprint smears of parathyroid glands obtained after parathyroidectomy, our study included direct smears of FNAB of parathyroid glands and this is probably the cause of the differences in these observations on Romanowsky stain between the two studies. Certain cellular architectural features can also be very helpful in identifying a parathyroid origin. On smears, the frequent presence of many naked nuclei in close association with adjacent clusters and the presence of a prominent vascular network with epithelial cells clinging to it may be useful clues. It is noteworthy that loose 2-dimensional clusters may have a rounded configuration and thus mimic thyroid macrofollicles and that tight small clusters of cells may resemble thyroid microfollicles.

Some authors have suggested that the characteristics of the smear's background maybe helpful in distinguishing cells of parathyroid origin from thyroid follicular cells [1,32]. Kini et al [32] and Abati et al. [1] concluded that colloid-like material and macrophages were specific to cells of thyroid origin, but we found these features in 12% and 18% respectively, of our parathyroid aspirates, respectively, none of which were localized in intrathyroidal, regions. These results are similar to those of earlier reports: Bondeson et al [25] reported colloid-like material and macrophages in 21% and 10% of lesions, respectively, and Tseng et al. [4] reported them in 24% and 9% of lesions, respectively.

Some studies have suggested that parathyroid hyperplasia can be distinguished from parathyroid adenoma based on cytomorphologic features [2-4,33]. Parathyroid hyperplasia was reported to exhibit more cellular monomophism [2,4] and arrangement in sheets, [33] while parathyroid adenoma has been described as showing a pleomorphic pattern with anisokaryosis [2-4] and microfollicular arrangements [33]. We disagree with those conclusions because we did not find characteristic distinguishing cytologic features for normal parathyroid glands and their tumors. Cytomorphologic features can be helpful in recognizing a parathyroid origin of cells, but they cannot be used to distinguish normal parathyroid from hyperplasia, adenoma, or carcinoma. Other investigators agree with us [1,27,34]. However, the diagnosis of parathyroid carcinoma can sometimes be made in the appropriate clinical setting in patients presenting with hypercalcaemia on the basis of results from FNAB of neck lesions, especially if the aspirate is composed of cells that are markedly pleomorphic, including bizarre giant cells with extreme cellularity; dyshesion, marked malignant anaplasia, abnormal mitotic figures and necrosis [9,10,35,36].

In addition to thyroid follicular cells, which are most commonly considered in the differential diagnosis of parathyroid glands and lesions, medullary thyroid carcinoma (MTC) can occasionally be confused with cells originating from the parathyroid gland, particularly in cases of recurrence or metastasis to the neck. MTC and parathyroid glands/lesions have a similar stippled nuclear chromatin. While MTC can also have round to oval cells, loose clusters, and single cells, the simultaneous presence of spindle and oval cells with a granular cytoplasm and an absence of naked nuclei usually favors a diagnosis of MTC. Familiarity with the patient's clinical history and use of appropriate ancillary immunostaining for calcitonin in difficult cases may help prevent the misdiagnosis of parathyroid glands/lesions for MTC and vice versa.

Immunocytochemical analysis is an extremely important ancillary tool for the definitive diagnosis of a lesion's parathyroid origin. All 14 of our cases in which smears were immunostained for PTH showed reactivity. It is to be noted that in 6 of these cases destained Pap smears were used for immunostaining for PTH with results that were comparable to those of cell block sections. Unlike us, other investigators have reported only equivocal staining in 30–40% of their cases [29,37]. It has been suggested that equivocal staining may be related to low storage of PTH in the cytoplasm [37]. Other immunohistochemical markers that may help exclude a thyroid origin of the cells (including MTC) include thyroglobulin, chromogranin, synaptophysin, and calcitonin.

The only case of metastatic parathyroid carcinoma in our study was to the liver. Parathyroid carcinoma is a rare malignancy and is an uncommon cause of PTH-dependent hypercalcemia [38]. The tumor is indolent and tends to recur locally at the operative site and spread to contiguous structures in the neck [38]. Metastasis can occur by means of both lymphatic and hematologic spread, with the cervical lymph nodes (30%) and lung (40%) being the most common sites for metastases, followed by the liver (10%) [38]. In our case of metastatic parathyroid carcinoma, FNAB exhibited a relatively bland morphology consisting of oval and spindle cells. The nuclear chromatin was granular to neuroendocrine in appearance, and the cytoplasm was fine and delicate. Because of the spindle cell morphology, other spindle cell lesions such as angiomyolipoma, smooth muscle tumors, and melanoma were included in the differential diagnosis. PTH immunostaining helped establish the diagnosis.

In conclusion, while US-guided FNAB is useful in confirming the diagnosis of clinically suspected parathyroid gland and lesions, in majority of cases it is utilized for detecting parathyroid glands in unexpected locations, such as in the thyroid bed or within the thyroid gland. Although there is significant overlap in the cytomorphologic features of cells derived from parathyroid and thyroid glands, the presence of stippled nuclear chromatin, usual presence of a vascular network with attached epithelial cells, and the frequent occurrence of single cells, including many naked nuclei, favor a parathyroid origin. The presence of colloid-like material and arrangement of cells as rounded loose clusters mimicking thyroid follicles can be significant pitfalls that may lead to the misdiagnosis of parathyroid glands/lesions to be derived from thyroid gland tissues. The distinction of the different parathyroid lesions including hyperplasia, adenoma and carcinoma cannot be made solely on the basis of cytologic features. Immunostaining for PTH can be performed on destained Pap smears or cell block sections with comparable results and can confirm a lesion's parathyroid origin.
